# RSV vaccination strategies for high-risk patients 2023: a collaborative position paper by leading German medical societies and organizations

**DOI:** 10.1007/s15010-023-02141-5

**Published:** 2023-12-07

**Authors:** Marylyn Addo, Oliver Cornely, Michael Denkinger, Georg Ertl, Susanne Herold, Mathias Pletz, Gernot Rohde, Tobias Welte, Wolfram Windisch, Martin Witzenrath

**Affiliations:** 1 German Center for Infection Research, University Medical Center Hamburg-Eppendorf, Hamburg, Germany; 2 German Society for Hematology and Oncology, University of Cologne, Faculty of Medicine, University Hospital Cologne, Cologne, Germany; 3 German Society for Geriatrics, University Hospital Ulm & Agaplesion Bethesda, Ulm, Germany; 4 German Society for Internal Medicine, University Hospital Würzburg, Würzburg, Germany; 5 German Society for Infectious Diseases, University Hospital Giessen-Marburg, Giessen, Germany; 6 Paul Ehrlich Society, University Hospital Jena, Jena, Germany; 7 Capnetz Foundation, University Hospital, Frankfurt a. M., Germany; 8 German Center for Lung Research, Hannover Medical School, Hannover, Germany; 9 German Respiratory Society, German Respiratory League, University Witten/Herdecke, Witten, Germany; 10 German Respiratory Society, Charité-Universitaetsmedizin Berlin, Berlin, Germany

**Keywords:** RSV (respiratory syncytial virus), Persons aged 60 years or older, Chronic lung disease, Effective vaccines, Severe RSV-associated respiratory infections, Recommendation

## Abstract

Respiratory syncytial virus (RSV) inflicts severe illness and courses of infections not only in neonates, infants, and young children, but also causes significant morbidity and mortality in older adults and in people with immunosuppression, hemato-oncologic disease, chronic lung disease, or cardiovascular disease. In June and August 2023, effective vaccines against RSV were approved for the first time by the European Medicines Agency (EMA) for the EU. The respective pivotal studies showed a very high efficacy of the vaccine in preventing severe RSV-associated respiratory infections. At this point, use of the respective vaccines is restricted to persons aged 60 years or older, according to the registration studies. We therefore recommend use of the vaccination in persons aged 60 years or older. In addition, we recommend use of the vaccination in adults of any age with severe pulmonary or cardiovascular pre-existing conditions, as well as in adults with significant immune compromise, after individual consultation with the treating physician. Cost coverage can be applied for individually with the responsible health insurance company.

## Background

Respiratory syncytial virus (RSV) causes a high burden of disease worldwide, which in studies has been comparable to influenza virus infections. RSV infections not only threaten newborns, infants, and young children, but can also cause severe disease progression and complications of pre-existing conditions in older and pre-diseased adults. RSV infections in adults are rarely detected in daily practice because, in contrast to influenza, no suitable point-of-care test is available. In 2015, an estimated 1.5 million cases of RSV-positive respiratory infections occurred in adults over 65 years of age in industrialized nations [1]. RSV infections occur seasonally in clusters during the winter months. A recent cohort study showed a further increase in the incidence of RSV infections after the first waves of the COVID-19 pandemic [2].

Albeit life-threatening courses of upper respiratory tract infections with RSV are rare, morbidity and mortality rates in people hospitalized for RSV pneumonia are comparable to those suffering from influenza and from pneumococcal pneumonia. People with malignant hematologic disease (e.g., leukemia, multiple myeloma), a previous history of pulmonary or cardiovascular disease, as well as immunosuppressed individuals (e.g., patients treated with systemic glucocorticoids, after organ transplantation, with neutropenia < 500/µl, lymphopenia < 200/µl, or immunoglobulin deficiency) are at particular risk for severe RSV infection. In addition to the risk of acute and severe respiratory infections, RSV infection also increases the risk of subsequent cardiovascular events, which can substantially increase morbidity and mortality particularly in those individuals with pre-existing cardiovascular risk factors and conditions.

The treatment strategy for acute RSV infection is primarily supportive. The monoclonal antibody nirsevimab has been approved since 2022 for passive immunization of infants to prevent RSV infection, but no specific therapies are currently available to treat acute RSV infection in adults [3].

Effective vaccines against RSV were approved for the first time by the European Medicines Agency (EMA) in June and August 2023 for the EU based on two large randomized controlled trials. The German Standing Commission on Vaccination (STIKO) is currently evaluating the data on these vaccines. Being the basis for vaccine-reimbursement considerations and therefore possible general availability in Germany, their recommendations are of central importance for public health.

## Results of randomized controlled trials on RSV vaccines

Glaxo Smith Kline’s RSV vaccine, marketed under the trade name Arexvy^®^, contains the adjuvant AS01E in addition to the viral prefusion-stabilized F protein. The vaccine was evaluated for efficacy, immunogenicity, and safety of a single dose of the vaccine in adults ≥ 60 years of age in the AReSVi-006 trial [4].

The RSV vaccine was administered to 12,464 adults aged 60 years and older, while 12,494 received placebo. Analysis after one RSV season showed vaccine efficacy of 82.6% for protection against RSV-associated lower respiratory tract disease, with 7 cases of disease after vaccination vs. 40 cases of disease in the placebo arm. Vaccine efficacy was 94.1%, for 1 case of severe disease after vaccination for protection against severe RSV-associated lower respiratory tract disease vs. 17 cases of severe disease in the placebo arm. Analysis after 2 RSV seasons showed vaccine effectiveness of 67.2% for protection against RSV-associated lower respiratory tract disease and 78.8% against severe RSV-associated lower respiratory tract disease.

Pfizer’s RSV vaccine, marketed under the trade name Abrysvo^®^, is bivalent and contains prefusion-stabilized F protein of RSV A and B, but no adjuvant. The vaccine was evaluated for efficacy, immunogenicity, and safety of a single dose of the vaccine in adults ≥ 60 years in the RENOIR trial [5]. The RSV vaccine was administered to 18,488 adults aged 60 years and older, while 18,479 received placebo. Interim analysis after one RSV season showed a vaccine effectiveness of 66.7% for protection against RSV-associated lower respiratory tract disease involving at least two signs or symptoms (cough, wheeze, sputum production, shortness of breath, or tachypnea) lasting longer than one day in 11 vs. 33 cases of illness. Vaccine effectiveness was 85.7% for 2 cases of illness in vaccinated patients for protection against RSV-associated lower respiratory tract disease with at least 3 signs or symptoms lasting longer than one day, with 14 cases of illness in the placebo arm.

Thus, a total of more than 30,000 vaccinated subjects were examined in the two studies. The results of both pivotal studies demonstrate the efficacy of both protein vaccines. Both vaccines caused adverse events in persons of 60 years and older, most of which resolved within a few days of onset and were comparable in frequency and intensity to vaccinations against other respiratory viruses [6, 7]. Six cases of inflammatory neurologic events were reported across the trials in older adults within six weeks after vaccination (i.e., three in the AReSVi-006 and three in the RENOIR trial) with the possibility of a causal relationship, compared with no cases within six weeks after placebo. In addition, no safety concerns were identified.

## Discussion

RSV infections are associated with a high burden of disease, particularly from complications of pre-existing underlying conditions. Now for the first time, effective vaccines for the prevention of RSV infection are available, and their high efficacy and safety have been demonstrated in two multicenter randomized trials. Widespread use of these vaccines could significantly reduce the direct burden of disease in the most at-risk populations, ameliorate individual disease trajectories, and help reduce the seasonal burden of acute respiratory infections on the healthcare system. Effective prevention of RSV infection may further help prevent serious pulmonary and cardiovascular complications.

To date, study data are only available for individuals aged 60 years and older, which is why approval of the vaccine is limited to this age group. In the presence of severe preexisting conditions associated with high risk from RSV infection, vaccination appears potentially reasonable outside of licensure in people younger than 60 years. This may encompass patients with malignant hematologic disease, a previous history of relevant pulmonary or cardiovascular disease, as well as severely immunosuppressed individuals who are at particular risk for severe RSV infection.

The disease burden of RSV infections due to indirect and delayed severe complications is often underestimated. With the newly licensed vaccines, effective and safe means of prevention are now available. It is our medical responsibility to provide the best possible protection for the high-risk patients we care for. With the winter season approaching and waves of infection expected, prevention using proven effective and safe vaccines should be a high priority. Therefore, vulnerable groups should be offered RSV vaccination in addition to influenza, COVID-19, and pneumococcal vaccination when appropriate. Either RSV vaccine can be given concurrently with a seasonal influenza vaccine.

Both vaccines have been available in German pharmacies (at a price of approximately €213.61 per single dose) since October 2023. For both vaccines, an individual application is currently (November 2023) required for cost coverage by the health insurance.

We also refer to the recommendation for RSV vaccination of the German Society for Hematology and Oncology:

### https://www.dgho.de/aktuelles/news/news/2023/download/rsv-impfung-20230815.pdf

(8197 characters with spaces/18000 recommended by Journal *infection).*

## Data Availability

Not applicable.
